# Call to Action for Enhanced Equity: Racial/Ethnic Diversity and Sex Differences in Stroke Symptoms

**DOI:** 10.3389/fcvm.2022.874239

**Published:** 2022-05-03

**Authors:** Floortje L. Hosman, Sabine Engels, Hester M. den Ruijter, Lieza G. Exalto

**Affiliations:** ^1^Department of Neurology, University Medical Center Utrecht Brain Center, University Medical Center Utrecht, Utrecht, Netherlands; ^2^Laboratory of Experimental Cardiology, University Medical Center Utrecht, Utrecht, Netherlands

**Keywords:** stroke, sex, intersectionality, ethnic diversity, symptoms

## Abstract

**Background:**

Fundamental aspects of human identity may play a role in the presentation of stroke symptoms and, consequently, stroke recognition. Strokes must be recognized and treated expeditiously, as delays result in poorer outcomes. It is known that sex plays a role in the presentation of symptoms, such that non-traditional symptoms are more commonly observed among women. However, factors such as geographical location and race/ethnicity, and the interactions between these various factors, need to be considered. This will provide an intersectional approach.

**Methods:**

A systematic review and meta-analysis of the literature was conducted to investigate differences in the presentation of stroke symptoms between sexes. Using PubMed and Embase, a search involving the components sex, symptoms and stroke was completed and yielded 26 full-text manuscripts.

**Results:**

Our findings indicate that there is substantial overlap in stroke symptom presentation in men and women. Nonetheless, some differences in the clinical manifestations of stroke were observed. In addition, it was discovered that only three studies were conducted outside of North America and Europe. Furthermore, only two studies reported symptoms based on both sex and racial/ethnic group.

**Conclusion:**

These findings indicate a research gap and call for increased research in order to uncover the possible interactions between sex and race/ethnicity in an intersectional approach. Resultantly, stroke recognition could be improved and greater equity in healthcare can be achieved.

## Introduction

Prompt stroke symptom recognition is of critical importance to prevent pre- and in-hospital delays (1). Minimizing these delays is crucial, as the efficacy of ischemic stroke treatment is time-dependent (2). Symptom presentation and, correspondingly, stroke recognition may depend on fundamental aspects of human identity. These aspects should not be considered independently, as they interact and are best described using the term intersectionality. Intersectionality describes the complex interconnectedness of elements such as gender, sex, race and ethnicity, with class, education, nationality and religion (3).

It is known that women are not only older by an average of 5 years when experiencing a stroke, but also suffer from more severe strokes (4). Risk factors and stroke subtypes differ between women and men, with women being more likely to suffer from atrial fibrillation and total anterior circulation syndrome or cardioembolic strokes (4, 5). On the other hand, men are more likely to be diagnosed with atherosclerosis and experience posterior circulation stroke or lacunar stroke (4, 5). Stroke subtypes are linked to symptom presentation, implying potential sex-based symptom differences. A recent systematic review on sex differences in stroke symptoms concluded that women are more likely to present with non-traditional stroke symptoms. Symptoms such as changes in level of consciousness, mental status change and headache are experienced more commonly by women (6).

Racial and ethnic group differences in risk factors and stroke subtypes exist, and potentially lead to additional stroke symptom disparities. Black populations carry a greater burden of stroke risk factors such as hypertension, obesity and diabetes mellitus (7). Diabetes mellitus is also a common risk factor in Hispanic populations (8). On the other hand, prevalence of large artery atherosclerosis is higher in Caucasians (9). Lacunar infarctions and transient ischemic attacks are more common among Blacks compared to Caucasians, whilst Blacks have lower odds of a cardioembolic stroke, which are more prevalent in Hispanic and Caucasian populations (9, 10). In addition to differing distributions of risk factors and stroke subtypes, a study in Northern America indicated in-hospital delays among ethnic minority groups. Blacks experience longer waiting times and additionally, ethnic minorities are less likely to receive thrombolysis as a treatment for acute ischemic stroke (10). Beliefs, attitudes and discrimination are likely to play a role in these delays in addition to a potential role of racial-ethnic symptom differences.

We conducted a systematic review and meta-analysis of the literature investigating differences in the presentation of stroke symptoms between women and men, and analyzed geographic diversity. Secondly, we aimed to identify studies that reported symptoms based on both sex and racial/ethnic group.

## Materials and Methods

### Search Strategy

Using PubMed and Embase, a search involving the components *sex, symptoms* and *stroke* and synonyms was conducted. The search query, last updated on January 18th, 2021, resulted in 21,304 hits in EMBASE and 10,411 in PubMed.

### Study Selection

A title-abstract screen followed by a full-text screen was performed. The screening procedure was completed by two independent reviewers (F.H. and S.E.). Debatable cases were discussed with L.E. to achieve consensus. Included articles focused on ischemic strokes or transient ischemic attacks, articles solely about hemorrhagic strokes or not about strokes were excluded. Included articles stated the quantitative results for symptom presentation differences between men and women. Only articles in Dutch or English were considered. Case studies, meta-analyses and systematic and literature reviews were excluded. Studies involving animals, patients under the age of 18 or only men/women were excluded from the review. The full-text screen excluded abstract-only congress papers and cases in which the full-text article was unavailable even after contacting the authors. A total of 282 articles were screened in the full-text screen, eventually yielding a total of 25 remaining papers. Snowballing allowed for the identification of an additional article, resulting in a total of 26 eligible studies in this systematic review and meta-analysis (Supplementary Material 1).

## Results

A total of 493,289 participants were involved in these studies, 50.1% of whom were women. The results of the meta-analysis indicate that there are sex differences in the presentation of stroke symptoms. Despite substantial overlap in stroke symptom presentation in women and men, some differences in the clinical manifestations of stroke were observed. Men have higher odds of presenting with the traditional symptoms of postural instability (OR 0.72; 95% CI, 0.59–0.88) and dysarthria (OR 1.03; 95% CI, 0.94–1.12). Women have higher odds of experiencing the traditional symptom aphasia (OR 1.18; 95% CI, 1.09–1.28). Non-traditional symptoms were found to be more common in women, who were shown to have increased odds of headache (OR 1.22; 95% CI, 1.04–1.43), mental status change (OR 1.24; 95% CI, 1.02–1.52), seizure/convulsions (OR 1.27; 95% CI 1.01–1.60), dysphagia (OR 1.33; 95% CI, 1.06–1.68), incontinence (OR 1.45; 95% CI, 1.32–1.59) and altered consciousness (OR 1.49; 95% CI, 1.32–1.68) (Supplementary Material 2).

**Figure 1:**
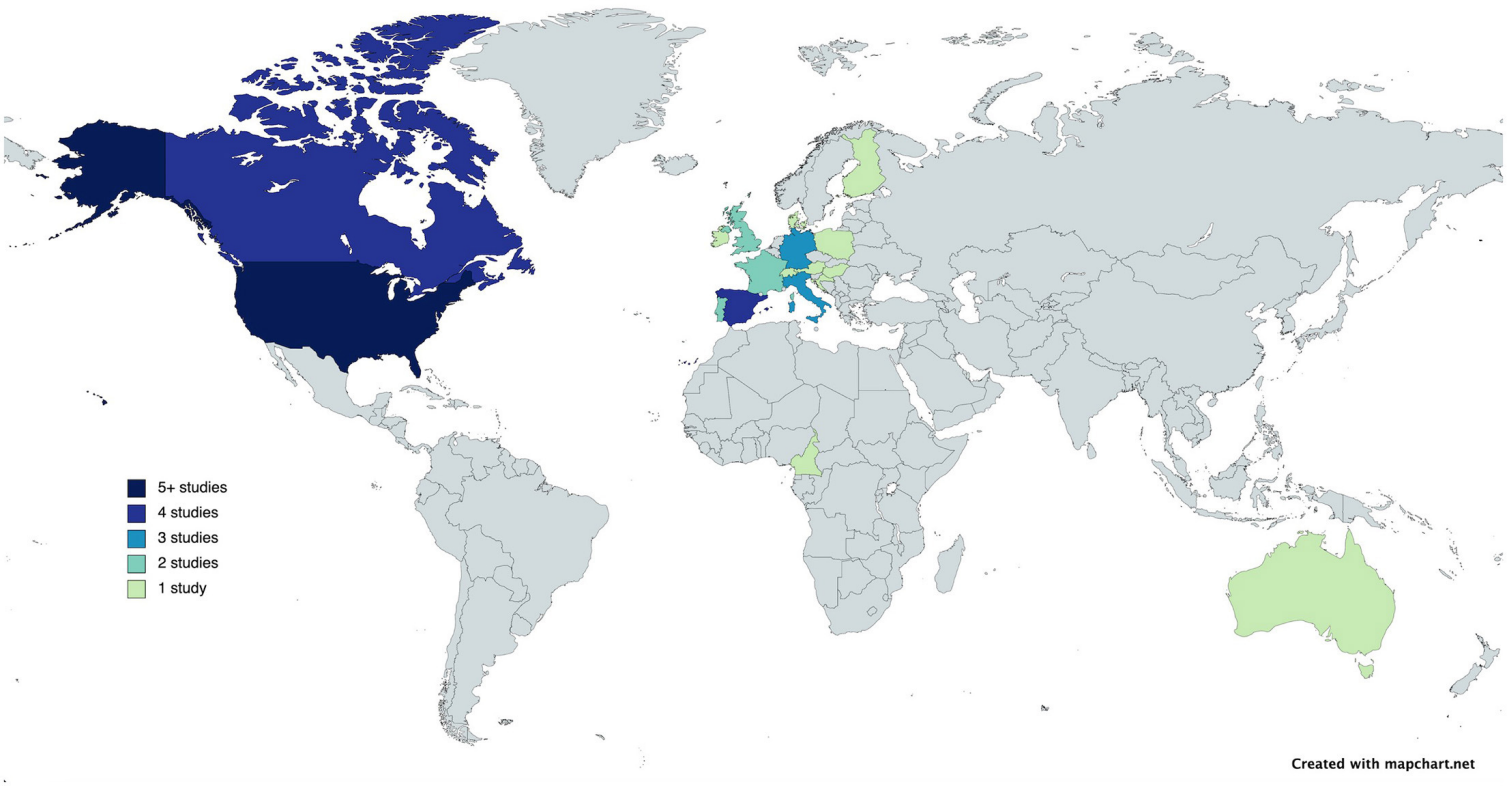
World map depicting the number of studies (color) on stroke symptoms in women and men were conducted.

Additional analyses of the geographical location of included studies allowed us to observe that only three of the 26 studies in this systematic review were conducted outside of North America or Europe (Figure 1). Out of the 26 studies, 14 reported the racial/ethnic distribution of the population whilst only two studies reported symptom presentation differences based on ethnicity (Table 1) (10, 33). These two studies included all stroke types and the results indicated that the frequency of stroke symptoms varies by sex as well as race/ethnicity. When evaluating differences between 226 Blacks and 248 Caucasians, Rathore et al. (33) found that Blacks are more likely to experience paresis of the face, arm or leg (78.2 vs. 85.4%; *p* = 0.044). Results also indicate that Caucasians are more likely to experience gait disturbance (13.3 vs. 8.0%; *p* = 0.061) and vertigo (3.2 vs. 0.9%; *p* = 0.077) (33). In line with this, Mochari-Greenberger et al. (17) (*n* = 398,798) found that, compared to Caucasians, weakness/paresis is more likely to be experienced by Blacks (OR 1.16; 95% CI, 1.14–1.19), as well as Asian (OR 1.23; 95% CI, 1.17–1.29) and Hispanic (OR 1.09; 95% CI, 1.06–1.12) populations. Mochari-Greenberger et al. (17) also more intricately examined the interaction between sex and racial/ethnic group and found that there were significant differences for the symptoms aphasia and altered consciousness. Namely, when compared to Caucasian men, aphasia is more common among Black men (OR 1.07; 95% CI, 1.04–1.10) and less common among Hispanic men (OR 0.95; 95% CI, 0.92–0.99). In women, Hispanics (OR 0.90; 95% CI, 0.87–0.94) and Asians (OR 0.91; 95% CI, 0.85–0.96) are less likely to experience this symptom compared to Caucasians. As for altered levels of consciousness, compared to Caucasian men, this symptom is more common among Black (OR 1.17; 95% CI, 1.13–1.20), Hispanic (OR 1.11; 95% CI, 1.06–1.16), and Asian (OR 1.07; 95% CI, 1.00–1.13) male populations. In women, however, presentation with this symptom does not differ significantly between Caucasians, Blacks, Hispanics, and Asians (17). It is important to note that both studies were conducted in the United States of America. Thus, more geographically diverse research is of interest. A meta-analysis looking at the intersection between sex and ethnicity in stroke symptom presentation was not feasible with only two studies.

**Table 1:** Baseline characteristics of all included studies.

**Study**	**Country**	**Study design**	**Collection years**	**Sample size** **(women/men)**	**Mean age** **(women/men)**	**Race/ethnicity**	**Stroke type**	**Main focus**
Medlin et al. (11)	Switzerland	Retrospective cohort study	2003–2016	3993 (1,761/2,232)	73.4 (77.0/69.9)	Baseline characteristics^*^ non-Caucasian	IS	Sex differences in acute ischemic stroke
Gocan et al. (12)	Canada	Retrospective cohort study	2015	671 (312/259)	67.3	Not mentioned	IS, TIA, HS	Sex differences in stroke symptoms and features
Yu et al. (13)	Canada	Multicenter prospective cohort study	2013-2017	1648 (770/878)	Median: 70 (71/69)	Baseline characteristics white/black/ Asian/ Aboriginal	IS, TIA	Sex differences in 1) presentation and 2) outcome of TIA or minor stroke
Zrelak et al. (14)	USA	Descriptive prospective study	2014–2016	59 (30/29)	66.9 (67.7/66.1)	Baseline characteristics white/black/ Asian	IS	Sex differences in stroke symptoms and care-seeking behavior
Mapoure et al. (15)	Cameroon	Hospital-based prospective cohort study	2010–2017	818 (363/455)	60 (62.9/58.4)	Study of black Africans	IS, HS	Sex differences in stroke
Li et al. (16)	Canada	Multicenter cohort study	2003–2008	5,991 (2,912/3,079)	Not specified	Not mentioned	TIA	Sex differences in TIA
Mochari-Greenberger et al. (17)	USA	Cohort study	2011–2014	398,798 (201,017/197,781)	Median: 71	Differences in symptoms based on ethnicity	IS, HS, undetermined	Sex and race differences in EMS transport of stroke patients
Chang et al. (18)	Sri Lanka	Population-based cross-sectional study	Not specified	24 (8/16)	63.7 (61.6/64.7)	Baseline characteristics Sinhalese	IS, HS	Sex differences in prevalence and risk factors of stroke
Madsen et al. (19)	USA	Multicenter study	2010	1991 (1,097/894)	Median: 74/67	Baseline characteristics black	IS	Sex differences in time to emergency department arrival
Acciarresi et al. (20)	Italy	Prospective cohort study	2005–2012	1,883 (811/1,072)	75.4/70.14	Not mentioned	IS, TIA, HS	Sex differences in stroke symptoms
Koh et al. (21)	Not specified	Online study	2010–2011	174 (91/83)	Divided into age groups	Not mentioned	IS, HS	Sex differences in stroke experiences
Arboix et al. (22)	Spain	Prospective hospital-based study	1986–2004	733 (310/423)	71.8/77.3	Not mentioned	IS	Sex differences in lacunar stroke
Kropp et al. (23)	Europe	Multicenter, multinational prospective observational study	Not specified	4,431 (1,801/2,630)	44.7 (43.4/45.7)	Not mentioned	IS, TIA	Headache as a symptom in stroke
Jerath et al. (24)	USA	Population-based study	1985–1989	449 (268/181)	75 (79/70)	Baseline characteristics white	IS	Sex differences in stroke symptoms and signs
Gall et al. (25)	Australia	Prospective population-based study	1996–1999	1,316 (731/585)	74 (76/72)	Not mentioned	IS, HS	Sex differences in stroke
Lisabeth et al. (1)	USA	Comparative study	2005–2007	461 (224/237)	Median: 67 (68/66)	Baseline characteristics white/black/ Asian/Pacific Islander	IS, TIA, undetermined	Sex differences in stroke symptoms
Gargano et al. (26)	USA	Comparative state-wide hospital-based study	2002	1,922 (1,041/881)	70 (72/68)	Baseline characteristics black/white/ other	IS, TIA, HS	Sex differences in stroke symptoms and emergency department delay
Stuart-Shor et al. (27)	USA	Comparative hospital-based study	1999–2004	1,107 (608/499)	73 (75.8/69.7)	Baseline characteristics white/black/ Asian/other/ unknown	IS	Sex differences in stroke symptoms (presenting and prodromal)
Foerch et al. (28)	Germany	Prospective country-wide hospital-based study	1999–2005	53,414 (26,319/27,095)	72.1 (75.3/69.1)	Not mentioned	IS, HS	Sex differences in acute stroke care of elderly patients
Barrett et al. (29)	USA	Prospective multicenter study	Not specified	505 (229/276)	Median: 65	Baseline characteristics black/white/ other	IS	Sex differences in stroke severity and symptoms
Kapral et al. (30)	Canada	Multicenter study	2001–2002	3,323 (1,527/1,796)	Median: 71 (73/69)	Not mentioned	IS, TIA, HS	Sex differences in stroke management and outcome
Roquer et al. (4)	Spain	Hospital-based study	1995–2002	1,581 (772/809)	71.6 (74.6/68.8)	Not mentioned	IS, HS	Sex differences in stroke
Di Carlo et al. (31)	Europe (England, France, Germany, Hungary, Italy, Portugal, Spain)	Prospective multicenter multinational hospital-based study	1993–1994	4,499 (2,260/2,239)	71.8 (74.5/69.2)	Not mentioned	IS	Sex differences in acute stroke presentation, resource use and 3-month outcome
Labiche et al. (32)	USA	Prospective observational study	1998–2000	1,124 (657/467)	71.4 (73.6/69.8)	Baseline characteristics white	IS, TIA, HS	Sex differences in stroke symptoms and delayed diagnosis
Rathore et al. (33)	USA	Cohort study	1987–1997	474 (224/250)	62.5	Differences in symptoms based on ethnicity (white/black)	IS, HS, undetermined	Characterization of stroke symptoms
Arboix et al. (34)	Spain	Hospital-based prospective study	1986–1995	2,000 (967/1,033)	75.1/69.8	Not mentioned	IS, TIA, HS, LS	Sex differences in stroke

*IS, ischemic stroke; TIA, transient ischemic attack; HS, hemorrhagic stroke; USA, United States of America; EMS, emergency medical service; NIHSS, National Institutes of Health Stroke Scale*.

* *Baseline characteristics indicates that the study provides information concerning how many participants were of a certain specified ethnicity, but the symptoms are not presented based on ethnicity*.

## Discussion

The majority of studies on sex differences in stroke presentation were conducted in North America and Europe. The meta-analysis indicates that there are some differences in the clinical manifestations of stroke between men and women. Namely, traditional symptoms such as postural instability and dysarthria are observed more frequently in men, whilst women are more likely to experience non-traditional symptoms such as headache, mental status change, seizure/convulsions, incontinence and altered consciousness. This is in line with previous findings (6). Furthermore, the results of this mini-review indicate that only two previous studies have reported stroke symptom presentation differences based on sex and race/ethnicity. Their results suggest that the frequency of certain stroke symptoms vary by sex as well as race/ethnicity. Only one study investigated the interaction between sex and racial/ethnic group, and found that there were significant differences for the symptoms aphasia and altered consciousness.

It is incongruous that the majority of research concerning stroke symptoms has been conducted in North America and western Europe, when across the globe, age-standardized stroke incidence is reported to be highest in northern Asia, followed by eastern Europe (35). Other sources indicate that the highest stroke burden is observed in sub-Saharan Africa (36). Increased longevity as well as changes in lifestyle and socio-demographic factors contribute to a trend of increasing stroke burden in low- and middle-income country settings (37, 38). In addition to geographical factors, race can also be linked to stroke incidence. Blacks have double the risk of stroke compared to Caucasians, and worse outcomes as well as higher mortality rates are observed among Black populations (39). The question of a conceivable role of race/ethnicity in symptom presentation is raised by these epidemiological patterns in combination with knowledge of the distribution of stroke risk factors and subtypes among races. Since research concerning stroke symptoms is largely based in western countries with mainly Caucasian populations, Caucasians are overrepresented in this field of research, whilst insights into racial-ethnic differences in symptom presentation could prove to be relevant in stroke recognition and, accordingly, outcome improvement.

The systematic review and meta-analysis is not without limitations. Firstly, the quality of the data on stroke symptoms is dependent on the methods used in individual studies. Prospective data capture, for example, may be considered more reliable than retrospective data capture. Additionally, distinct data collection methods used in the included studies, such as medical record review and interviews, subsequently lead to different types of bias such as misclassification bias and recall bias, respectively. Secondly, multiple stroke symptoms can be present in one patient. It is not clear how often patients with stroke show isolated non-traditional symptoms—in other words, non-traditional symptoms that are not seen in combination with traditional symptoms. Furthermore, symptom frequency may be underreported due to a lack of recognition, especially for non-traditional symptoms, which could result in publication bias. Lastly, the data does not indicate the extent to which a symptom was experienced: whether it was mild or severe. Nor which symptom is most burdensome in terms of post-stroke disability. This is all relevant data within the scheme of stroke recovery.

Racial-ethnic and sex/gender stratification of stroke symptom presentation is necessary to deepen our understanding of symptom presentation, subsequently improving recognition. Improved recognition is directly associated with improved stroke outcomes (1). Recognition does not only entail recognition by the general public, but also by front line health workers and hospital staff. Although focus should remain upon the recognition of traditional symptoms, additional educational intervention programs or public health campaigns concerning racial-ethnic and sex/gender disparities in stroke symptoms may be necessary. However, the big picture is more complex. Stroke recognition is tied to social and environmental determinants. Here, there is room for intersectional research, investigating ethnicity and gender-based social constructions, as well as prejudice and discrimination, and how this affects stroke recognition. Limited recognition of the importance of intersectional research can be attributed to the reliance upon reductionist frameworks. Ultimately, research in this area and tackling underlying risk factors can aid the journey toward health equity in underrepresented populations. Lastly, improvements in the reporting of non-traditional symptoms are necessary to gain a more complete understanding of the sex and gender differences in this area, as well as potential racial-ethnic dissimilarities, and interactions between the two.

## Author Contributions

FH and SE: acquisition of data. FH: analysis of data and writing the first draft. HR and LE: conception and design. All authors contributed to the article and approved the submitted version.

## Funding

LE is WP leader sex differences in the Heart-Brain Connection Consortium, which was supported by the Netherlands CardioVascular Research Initiative: the Dutch Heart Foundation (CVON 2018-28 & 2012-06 Heart Brain Connection), Dutch Federation of University Medical Centers, the Netherlands Organization for Health Research and Development, and the Royal Netherlands Academy of Sciences. LE is recipient of 2020 Prize for Best Integration of Sex and Gender Considerations in a Cardiovascular Research Project, made available by Libin International Trainee Symposium: Research is Better with Sex and Gender!

## Conflict of Interest

The authors declare that the research was conducted in the absence of any commercial or financial relationships that could be construed as a potential conflict of interest.

## Publisher's Note

All claims expressed in this article are solely those of the authors and do not necessarily represent those of their affiliated organizations, or those of the publisher, the editors and the reviewers. Any product that may be evaluated in this article, or claim that may be made by its manufacturer, is not guaranteed or endorsed by the publisher.
