# Supplementary material for: Call to Action for Enhanced Equity: Racial/Ethnic Diversity and Sex Differences in Stroke Symptoms
Source: Front Cardiovasc Med. 2022 May 3;9:874239. doi: 10.3389/fcvm.2022.874239 (PMC9110690; doi:10.3389/fcvm.2022.874239)
Supplement: Supplementary file 1 [file Data_Sheet_1.docx]

Supplementary Material

**Supplement 1**

*Flow chart depicting the study selection process*


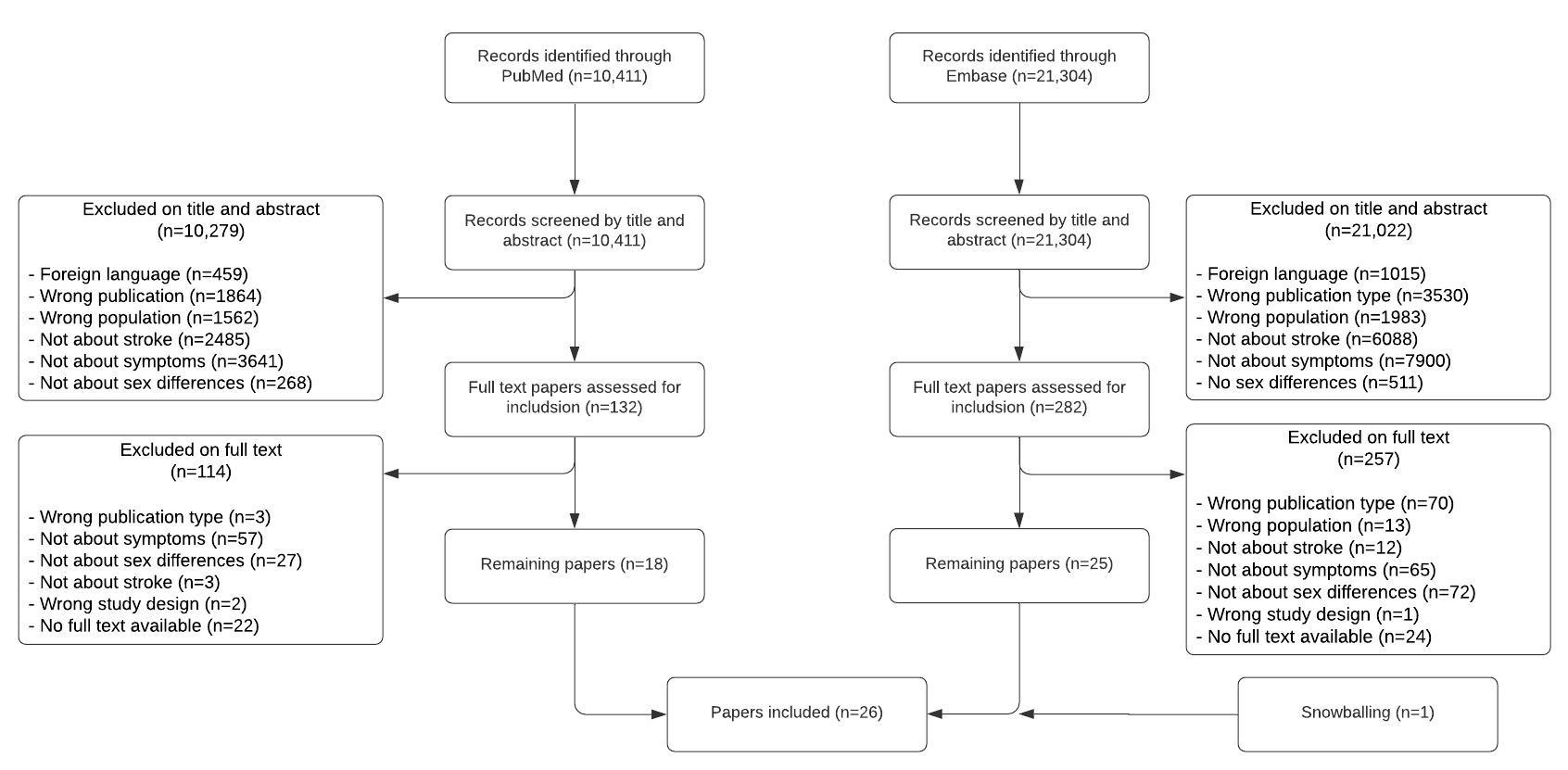


**Supplement 2**

*Odds ratio under the random effects model per symptom, representing symptoms experienced by women relative to men*

| Symptom | Studies (n) | Participants (n) | Women (%) / Men (%) | Odds ratio using random effects model | I^2^ |
| --- | --- | --- | --- | --- | --- |
| Traditional Symptoms | | | | | |
| Postural instability | 8 | 6532 | 16.45 / 21.17 | 0.72 [0.59-0.88] | 19.99% |
| Diplopia | 4 | 1442 | 4.03 / 5.02 | 0.74 [0.13-4.25] | 71.47% |
| Ataxia | 8 | 6518 | 12.93 / 13.30 | 0.77 [0.55-1.08] | 53.78% |
| Dizziness/vertigo | 12 | 11785 | 11.21 / 14.17 | 0.83 [0.69-1.01] | 53.43% |
| Dysarthria | 12 | 21515 | 33.21 / 35.61 | 0.88 [0.79-0.97] | 47.50% |
| Numbness/paresthesia | 15 | 24806 | 34.82 / 36.04 | 0.93 [0.83-1.04] | 59.43% |
| Visual field deficit/ loss | 16 | 21864 | 16.41 / 16.42 | 1.01 [0.88-1.15] | 45.46% |
| Weakness/paresis | 20 | 483269 | 67.27 / 67.99 | 1.03 [0.94-1.12] | 81.04% |
| Facial/eyelid droop | 3 | 4694 | 6.52 / 5.21 | 1.14 [0.61-2.16] | 19.83% |
| Aphasia | 14 | 422359 | 41.48 / 40.10 | 1.18 [1.09-1.28] | 61.46% |
| Hemianopia | 5 | 4579 | 31.68 / 17.31 | 1.79 [0.81-3.95] | 89.98% |
| Non-traditional symptoms | | | | | |
| Breathing problems | 3 | 2156 | 3.75 / 3.95 | 0.92 [0.39-2.14] | 0.00% |
| Nausea/vomiting | 5 | 4105 | 15.20 / 14.38 | 1.04 [0.62-1.73] | 54.50% |
| Lightheadedness | 4 | 2342 | 10.58 / 9.86 | 1.08 [0.77-1.50] | 0.00% |
| Pain | 8 | 6508 | 6.87 / 6.04 | 1.11 [0.79-1.56] | 30.30% |
| Headache | 15 | 25577 | 23.59 / 19.23 | 1.22 [1.04-1.43] | 76.48% |
| Mental status change | 10 | 17160 | 18.85 / 16.65 | 1.24 [1.02-1.52] | 59.95% |
| Seizure/convulsions | 6 | 5927 | 3.69 / 3.20 | 1.27 [1.01-1.60] | 0.00% |
| Dysphagia | 7 | 15295 | 18.40 / 15.01 | 1.33 [1.06-1.68] | 70.19% |
| Incontinence | 4 | 7015 | 34.31 / 27.48 | 1.45 [1.32-1.59] | 0.00% |
| Fatigue/drowsiness | 3 | 3204 | 10.87 / 7.45 | 1.46 [0.96-2.22] | 0.00% |
| Altered consciousness | 12 | 468364 | 24.45 / 19.67 | 1.49 [1.32-1.68] | 94.97% |
